# Complement-independent retinal pathology produced by intravitreal injection of neuromyelitis optica immunoglobulin G

**DOI:** 10.1186/s12974-016-0746-9

**Published:** 2016-10-20

**Authors:** Christian M. Felix, Marc H. Levin, Alan S. Verkman

**Affiliations:** 1 Department of Ophthalmology, University of California, San Francisco, San Francisco, CA USA; 2 Departments of Medicine and Physiology, University of California, San Francisco, 1246 Health Sciences East Tower, San Francisco, CA 94143-0521 USA; 3 Department of Ophthalmology, The Palo Alto Medical Foundation, Palo Alto, CA USA

**Keywords:** NMO, Retina, Müller cells, Aquaporin-4, Complement, Autoimmunity

## Abstract

**Background:**

Neuromyelitis optica (NMO), an autoimmune inflammatory disease of the central nervous system, is often associated with retinal abnormalities including thinning of the retinal nerve fiber layer and microcystic changes. Here, we demonstrate that passive transfer of an anti-aquaporin-4 autoantibody (AQP4-IgG) produces primary retinal pathology.

**Methods:**

AQP4-IgG was delivered to adult rat retinas by intravitreal injection. Rat retinas and retinal explant cultures were assessed by immunofluorescence.

**Results:**

Immunofluorescence showed AQP4-IgG deposition on retinal Müller cells, with greatly reduced AQP4 expression and increased glial fibrillary acidic protein by 5 days. There was mild retinal inflammation with microglial activation but little leukocyte infiltration and loss of retinal ganglion cells by 30 days with thinning of the ganglion cell complex. Interestingly, the loss of AQP4 was complement independent as seen in cobra venom factor-treated rats and in normal rats administered a mutated AQP4-IgG lacking complement effector function. Exposure of ex vivo retinal cultures to AQP4-IgG produced a marked reduction in AQP4 expression by 24 h, which was largely prevented by inhibitors of endocytosis or lysosomal acidification.

**Conclusions:**

Passive transfer of AQP4-IgG results in primary, complement-independent retinal pathology, which might contribute to retinal abnormalities seen in NMO patients.

## Background

Neuromyelitis optica (NMO) is an autoimmune inflammatory disease of the central nervous system (CNS) that causes optic neuritis and transverse myelitis, leading to loss of vision and motor function, reviewed in [1–4]. Affected tissues in NMO generally show astrocyte damage with complement activation, inflammatory cell infiltration, and demyelination. Most NMO patients are seropositive for immunoglobulin G autoantibodies against aquaporin-4 (AQP4), a water channel expressed on astrocytes. Human NMO pathology and rodent models of NMO produced by passive transfer of anti-AQP4 autoantibody (AQP4-IgG) suggest a pathogenesis mechanism in which AQP4-IgG binding to AQP4 causes primary complement- and cell-mediated astrocyte toxicity, with a secondary inflammatory response leading to oligodendrocyte injury, demyelination, and axon loss.

NMO patients often manifest retinal abnormalities including thinning of the retinal nerve fiber layer (RNFL) and ganglion cell layer (GCL) following episodes of optic neuritis [5–7]. During acute episodes of NMO optic neuritis, the inner nuclear layer (INL) typically thickens [8, 9], often coincident with inner retinal microcystic changes [10, 11]. It has been speculated that retinal injury in NMO may in part be a primary disease manifestation, although it has been difficult to resolve direct retinal injury from retrograde axon degeneration due to retrobulbar optic neuritis. These retinal abnormalities are also seen, albeit at lower frequency, following optic neuritis in multiple sclerosis and a variety of severe noninflammatory optic neuropathies [12–15].

The cellular makeup and structure of the retina is quite different from the optic nerve, spinal cord, and brain, where there are myelinated nerve fibers and AQP4 expression on astrocytes [16]. In the retina, AQP4 is expressed in two types of retinal glia: Müller cells, which are a specialized form of radial glia spanning the retinal inner limiting to outer limiting membranes, with the cell body lying within the INL, and astrocytes, which are mostly localized in the RNFL along with nonmyelinated axons of retinal ganglion cells (RGCs).

Here, to investigate the possibility that NMO autoantibodies could produce primary retinal injury, we exposed rat retinas to AQP4-IgG in vivo by intravitreal injection. Rats were chosen because they have human-like serum complement activity [17] and have been used in various models in which AQP4-IgG produces characteristic NMO pathology in CNS tissues [18, 19]. We found that intravitreally delivered AQP4-IgG efficiently bound to AQP4 on retinal Müller cells and, unexpectedly, produced unique complement-independent retinal injury, which was further characterized by studies on ex vivo retinal cultures exposed to AQP4-IgG.

## Methods

### Rats

Adult male Sprague-Dawley rats (age 8–10 weeks) were purchased from Charles River Laboratories (Wilmington, MA). CD59^−/−^ rats were generated using CRISPR-Cas9 technology, which will be reported separately. Protocols were approved by the University of California San Francisco Committee on Animal Research and were in compliance with the ARVO Statement for the Use of Animals in Ophthalmic and Visual Research.

### Intravitreal injections

Rats were anesthetized using 3 % isoflurane and topical proparacaine (0.5 %, Akorn, Lake Forest, IL), and their pupils were dilated with phenylephrine (2.5 %, Paragon BioTeck Inc., Portland, OR) and atropine (1 %, Akorn). In each eye, a full-thickness track was made through superotemporal sclera at the pars plana with a 30-gauge needle for intravitreal delivery of a 4-μL solution volume using a 33-gauge beveled needle attached to a 10-μL Hamilton syringe (Reno, NV). Microscopic examination was performed to confirm the absence of vitreous hemorrhage or gross lens trauma. Lubricating ophthalmic ointment was applied to protect the cornea until recovery from anesthesia.

Both eyes in each rat received the same treatment to control for potential contralateral effects of a given treatment. The following experimental groups were evaluated: (i) no injection, or injection of (ii) saline; (iii) control human IgG (control-IgG; 40 μg; Pierce Biotechnology, Rockford, IL); (iv) purified human monoclonal recombinant AQP4-IgG (rAb-53, 40 μg, ref. [20]); (v) recombinant monoclonal anti-AQP4 “aquaporumab”-lacking effector functions (AQP4-IgG^-CDC^, 4 μg, ref. [21]); or (vi) lipopolysaccharide (LPS) from *Escherichia coli* (5 μg; Sigma-Aldrich, St. Louis, MO). In some experiments, rat complement was inactivated by intraperitoneal injection of cobra venom factor (CVF; 600 U/kg, Quidel Corporation, Santa Clara, CA) 24 h before and 48 h after intravitreal injection of AQP4-IgG, as described in [19]. Rats were sacrificed 6 h, 24 h, 5 days, or 30 days after intravitreal injection. Globes were enucleated after transcardiac perfusion with phosphate-buffered saline (PBS) followed by 4 % paraformaldehyde, fixed for 4 h and left overnight at 4 °C in 30 % sucrose. The eyes were embedded in OCT and sectioned axially at 20-μm thickness.

### Retinal explant cultures

Rats were deeply anesthetized with isoflurane and then decapitated. The freshly enucleated eyes were immersed in ice-cold Hank’s balanced salt solution (HBSS) containing 1 % penicillin-streptomycin. Using a dissecting microscope, a circumferential incision was made at the pars plana, followed by removal of the anterior segment, lens, and vitreous body. With Dumont forceps, the retinas were separated from the sclera and separated from the optic nerve head. Each retina was cut radially and separated into four quadrants, which were each transferred with inner retinal surfaces facing up onto 12-mm-diameter filters (0.4-μm pore; Sigma-Aldrich) in 12-well plates.

Retinal explants were maintained immersed in a thin layer of serum-free culture medium at an air/medium interface in a 5 % CO_2_ incubator at 37 °C. Culture media contained neuronal growth medium (Neurobasal A) supplemented with 2 % B27, 1 % N2, L-glutamine (0.8 mM), and 1 % penicillin-streptomycin. One half of the media was replaced after 24 h in culture. AQP4-IgG (final 20 μg/mL) was added to some wells after the initial 24 h in culture. Some explants were also incubated with dynasore hydrate (inhibitor of dynamin-dependent endocytosis; 50 μM) or chloroquine (inhibitor of lysosomal degradation; 10 μM). At 24 h later, explants were fixed in 4 % PFA for 24 h and then placed in 30 % sucrose for 24 h at 4 °C before embedding in OCT. Sections were cut at 10-μm thickness perpendicular to the full-thickness retina.

### Immunofluorescence

Frozen sections were incubated in blocking solution (PBS, 1 % bovine serum albumin, 0.2 % Triton X-100) for 1 h prior to overnight incubation (4 °C) with primary antibodies against the following: AQP4 (1:200, Santa Cruz Biotechnology, Santa Cruz, CA), glial fibrillary acidic protein (GFAP, 1:100, Millipore), glutamine synthetase (GS, 1:500, Sigma-Aldrich), Brn3a (1:100, Santa Cruz Biotechnology), ionized calcium-binding adaptor molecule-1 (Iba1; 1:1000; Wako, Richmond, VA), C1q (1:50, Abcam, Cambridge, MA), or C5b-9 (1:50, Hycult Biotech, Uden, Netherlands), followed by appropriate species-specific Alexa Fluor-conjugated secondary antibody for 1 h at room temperature (1:200, Invitrogen, Carlsbad, CA). Rinsed sections were mounted with VECTASHIELD with 4’,6-diamidino-2-phenylindole (DAPI) (Vector Laboratories, Burlingame, CA). Staining with hematoxylin and eosin (H&E) was done using standard procedures. Sections were visualized on a Leica epifluorescence microscope (Wetzlar, Germany) or Nikon confocal fluorescence microscope (Melville, NY).

AQP4, GFAP, and Iba1 immunofluorescence were quantified in ×20 fields of central retina, 50 μm from the optic nerve head. AQP4 and GFAP fluorescence were defined using the polygon drawing tool and quantified using ImageJ (NIH, Bethesda, MD). For AQP4 quantification, retinal layers were segmented into RNFL + GCL, inner plexiform layer (IPL) + INL, and outer plexiform layer (OPL) + outer nuclear layer (ONL). GFAP was measured in two segments: (RNFL + GCL) and (IPL + INL + OPL + ONL). Data are presented as a percentage of area of immunofluorescence loss normalized to untreated retinas.

RGCs were counted at day 30 after intravitreal injection of AQP4-IgG or control-IgG as the density of Brn3a-positive nuclei in fluorescence micrographs of retinal flat mounts. After transcardiac perfusion, the eyes were enucleated and the retinas were removed and immunostained in culture wells on a shaker. Four radial relaxing incisions were made, and the retinas were flattened and coverslipped with VECTASHIELD mounting media. A total of 12 nonoverlapping images (×20 magnification), each including nonoverlapping posterior, middle, or anterior retina of one quadrant, were taken using the epifluorescence microscope. Brn3a-positive nuclei were counted semi-automatically using cell-counting plugins from ImageJ software.

### Optical coherence tomography and fundoscopy

Rats were induced and maintained under isoflurane gas anesthesia, and the eyes were numbed and dilated as before. The corneas were lubricated with 2.5 % Goniovisc (Accutome Inc., Malvern, PA) for direct contact with the imaging lens. The eyes were examined using the Micron III retinal imaging system (Phoenix Research Labs, Pleasanton, CA), and raw fundus photographs were captured. Spectral-domain optical coherence tomography (SD-OCT) horizontal line scans were acquired on a micron image-guided SD-OCT system (Phoenix Research Labs) by averaging 10 scans. Potential inner retinal atrophy was evaluated in vivo as longitudinal changes in thickness of the ganglion cell complex (GCC, encompassing RNFL, GCL, and IPL). GCC and total retinal thicknesses at single points 1500 μm from the nasal and temporal optic nerve head margins were averaged as a single point per eye.

### Statistics

Data are presented as mean ± S.E.M. The two-sided Student’s *t* test was used for direct comparisons between two means. When there were three or more groups, analysis was done using a one-way ANOVA with Dunnett post hoc test. Analysis was performed using Prism 5 GraphPad Software package (GraphPad Software, San Diego, CA). Significance levels were set at *p* < 0.05 (*) and *p* < 0.01 (**).

## Results

### Intravitreal AQP4-IgG reduces Müller cell AQP4 expression and causes reactive gliosis

**Fig. 1:**
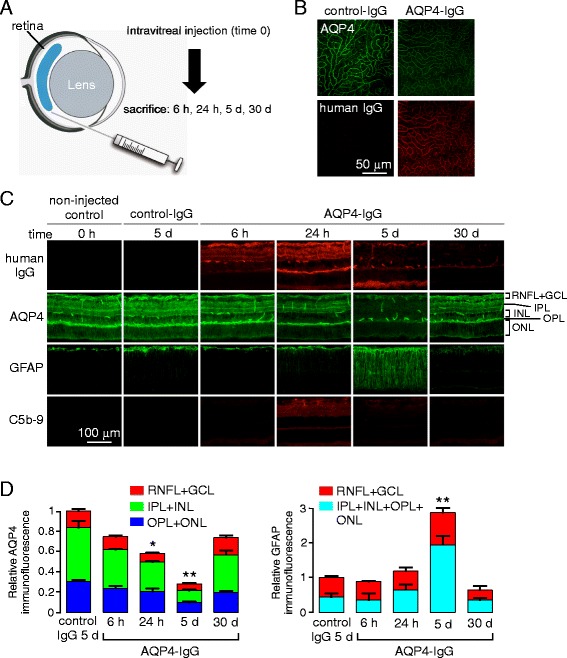
Consequences of intravitreal administration of AQP4-IgG in rat. **a** Injection approach. **b** AQP4 and human IgG immunofluorescence in retinal flat mounts 5 days after intravitreal delivery of 40 μg control human IgG or AQP4-IgG in rat. **c** Immunofluorescence on transverse retinal sections for experiments as in (**b**). Representative of 4 eyes per condition. **d** Relative immunofluorescence of AQP4 (*left*) and GFAP (*right*) in indicated retinal layers from sections as in (**c**) (mean ± S.E.M, 2 rats, 4 eyes per condition, *p < 0.05, **p < 0.01)

As diagrammed in Fig. 1a, rats were administered AQP4-IgG (or control human IgG) by intravitreal injection and then sacrificed at different times. The injected eyes were grossly normal at sacrifice, without uveitis or hemorrhage, and only rare focal lens opacifications. En face immunofluorescence of retinal flat mounts showed AQP4-IgG deposition on the retina in a membrane pattern at 3 days after injection, as seen using a secondary antibody against human IgG, with loss of AQP4 immunofluorescence (Fig. 1b). Human IgG binding was not seen in astrocytes along myelinated optic nerves (not shown), demonstrating selective exposure of the retina to AQP4-IgG.

Immunofluorescence of frozen sections of the posterior retina showed AQP4-IgG localization in a pattern corresponding to Müller cells (Fig. 1c). There was some loss of AQP4 immunofluorescence by 6 h, which became more marked at 24 h and 5 days but largely returned to initial levels by 30 days. Also notable was Müller cell gliosis, seen as an upregulation of GFAP expression most evident at 5 days, as well as a deposition of activated complement in an apparent perivascular pattern seen best at 24 h and 5 days. Quantification of retinal AQP4 and GFAP immunofluorescence is summarized in Fig. 1d.

**Fig. 2:**
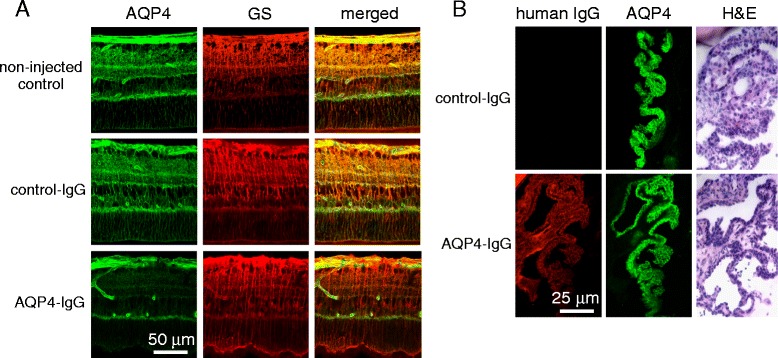
Selective loss of Müller cell AQP4 following intravitreal injection of AQP4-IgG. **a** AQP4 and GS immunofluorescence at 5 days after intravitreal AQP4-IgG injection under conditions as in Fig. 1c, visualized by confocal microscopy using a ×60 oil immersion lens. **b** Human IgG and AQP4 immunofluorescence and H&E staining of the ciliary body at 5 days following intravitreal AQP4-IgG injection. Images representative of four eyes

At higher magnification, AQP4 immunofluorescence colocalized with the Müller cell marker GS (Fig. 2a). GS immunofluorescence was stable even after AQP4 was greatly reduced, suggesting that the Müller cells remained viable. As expected, intravitreally delivered AQP4-IgG also localized to cell membranes of nonpigmented ciliary epithelia, the other major ocular site of AQP4 expression (Fig. 2b). In contrast to the retina, AQP4-IgG deposition in the ciliary body did not cause loss of AQP4 at 5 days after administration nor was there demonstrable inflammatory infiltrates as seen by H&E staining.

### Loss of Müller cell AQP4 following intravitreal AQP4-IgG is complement independent

**Fig. 3:**
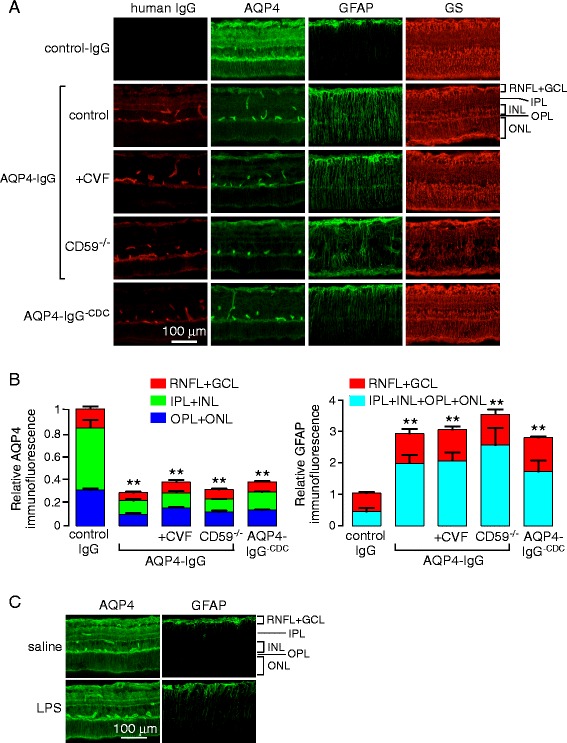
Complement-independent loss of Müller cell AQP4 following intravitreal AQP4-IgG administration. **a** Immunofluorescence of the central retina at 5 days after intravitreal administration of AQP4-IgG (or control-IgG). Where indicated, rats were treated with cobra venom factor (*CVF*) to inactivate complement or administered AQP4-IgG^-CDC^ (AQP4-IgG lacking complement effector function) in place of AQP4-IgG. In some studies, CD59^−/−^ rats were administered AQP4-IgG. **b** Relative AQP4 (*left*) and GFAP (*right*) immunofluorescence in indicated retinal layers from sections as in (**c**) (mean ± S.E.M, 2 rats, 4 eyes per condition, *p < 0.05, **p < 0.01). **c** AQP4 and GFAP immunofluorescence at 5 days after intravitreal injection of saline control (*top*) or 5 μg LPS (*bottom*)

A large body of data in human NMO and in animal models implicates complement deposition and activation on AQP4-expressing astrocytes in the brain and spinal cord in disease pathogenesis. To investigate whether complement activation is required to produce AQP4 loss following intravitreal AQP4-IgG injection, studies were done in rats treated with CVF, which inactivates complement and was shown previously to prevent NMO pathology following intracerebral injection of AQP4-IgG in rats [19]. At 5 days following intravitreal injection of AQP4-IgG, the CVF-treated rats showed similar loss of Müller cell AQP4 as seen in the untreated rats, as well as comparable Müller cell gliosis (Fig. 3a), suggesting that these retinal changes are complement independent. Also in support of this conclusion was the finding that delivery of an engineered AQP4-IgG lacking complement effector function (in place of AQP4-IgG) produced similar AQP4 loss and Müller cell gliosis. Moreover, exposure of the retinas of transgenic rats lacking the complement regulator protein CD59 to AQP4-IgG produced similar changes in AQP4 and GFAP immunofluorescence as in wildtype rats. The quantification of AQP4 and GFAP immunofluorescence is summarized in Fig. 3b.

To show that the loss of Müller cell AQP4 is specific to AQP4-IgG binding rather than a nonspecific effect of ocular inflammation, retinas were examined at 5 days after intravitreal delivery of LPS, a model that produces uveitis with vitritis and associated Müller cell gliosis [22]. Figure 3c shows preservation of Müller cell AQP4 despite inflammation with panuveitis and abundance of macrophages in the vitreous, Müller cell gliosis, and cellular infiltration (see below).

### Intravitreal AQP4-IgG produces retinal microglial activation with minimal leukocyte infiltration

**Fig. 4:**
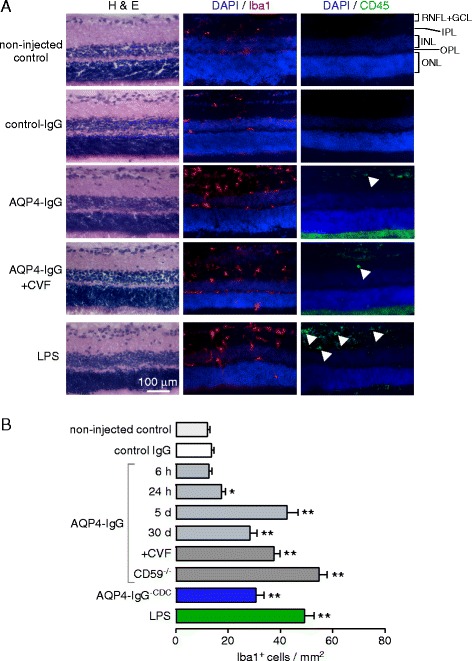
Retinal inflammatory response to intravitreal AQP4-IgG. **a** H&E staining, and Iba1 and CD45 immunofluorescence (with DAPI counterstain), in posterior retinas at 5 days after intravitreal injection of 40 μg AQP4-IgG (or control-IgG). *White arrowheads* indicate CD45^+^ cells. **b** Iba1^+^ cells per mm^2^ of full-thickness retina (mean ± S.E.M, 2 rats, 4 eyes per condition, *p < 0.05, **p < 0.01)

Iba1 is a marker of monocytes, including brain-derived microglia and infiltrative macrophages. Immunofluorescence showed a significant increase in the number of microglia throughout the inner retinal layers, especially in IPL, 5 days after intravitreal AQP4-IgG administration. Increased numbers of Iba1^+^ cells were also seen in CVF-treated rats, again supporting a complement-independent proinflammatory mechanism (Fig. 4a). As a positive control, intravitreal LPS increased the number of Iba1^+^ cells. Examination of individual cells at high magnification showed a characteristic stellate appearance indicative of microglial activation (Fig. 4b). Though resident microglia were activated by AQP4-IgG, comparatively few CD45^+^ leukocytes were seen in the retinas (Fig. 4a); as a control, abundant CD45^+^ infiltrates were seen in the retinas of the rats following intravitreal LPS. The bright green fluorescence at the bottom of the section (below the ONL) is due to nonspecific secondary antibody binding to the sclera, which occasionally remained attached to the retina during sectioning.

### AQP4-IgG produces loss of retinal ganglion cells

**Fig. 5:**
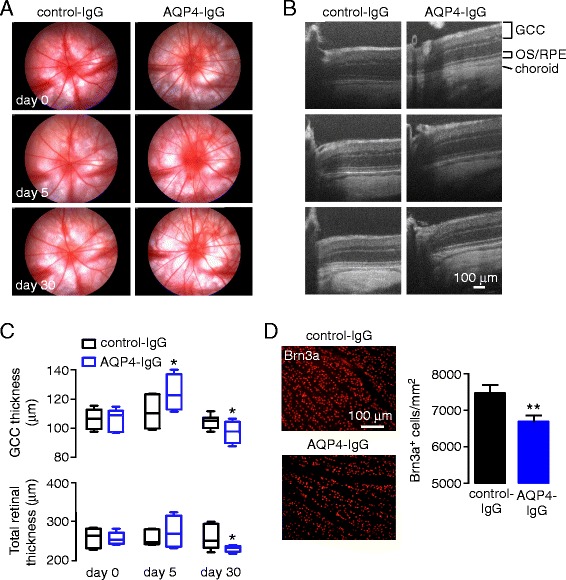
Fundoscopy, OCT, and RGC count in rat eyes following intravitreal injection of AQP4-IgG. **a** Serial fundus photographs of individual rat eyes following intravitreal injection of AQP4-IgG (or control-IgG). **b** SD-OCT horizontal line scans through the optic nerve head in rat eyes for studies as in (**a**). *OS/RPE* outer segment/retinal pigment epithelium. **c** GCC (*top*) and total retinal (*bottom*) thicknesses before and at 5 and 30 days after intravitreal injection of AQP4-IgG (or control-IgG) (*box and whisker plot with top line* indicating maximum value, *middle bar* the mean, and *lower line* the minimum, mean ± S.E.M, 3 rats, 6 eyes per condition, *p < 0.05, **p < 0.01). **d** (*Left*) Brn3a immunofluorescence in mid-peripheral retina in flat mounts at 30 days after intravitreal injection of AQP4-IgG or control-IgG. (*Right*) Brn3a^+^ RGC cell counts (mean ± S.E.M, 2 rats, 4 eyes per condition, *p < 0.05)

Retinal structures were examined longitudinally by in vivo fundus imaging. Fundus photography did not reveal acute vascular or inflammatory pathology, including the absence of retinal vascular occlusions, retinitis, or papillitis (Fig. 5a). OCT showed inner retinal thickening in the absence of microcyst formation at day 5 at which maximal AQP4 loss and microglial activation occurs, with mild though significant GCC and total retinal thinning at day 30 (Fig. 5b, c). As GCC layer thinning is consistent with loss of RGCs and/or Müller cell somas in the INL, Brn3a^+^ RGCs were quantified in whole retinal mounts at day 30. RGC density was significantly reduced in the AQP4-IgG- compared with control-IgG-treated eyes (Fig. 5d).

### Mechanism of AQP4-IgG-induced loss of Müller cell AQP4 studied in retinal explant cultures

**Fig. 6:**
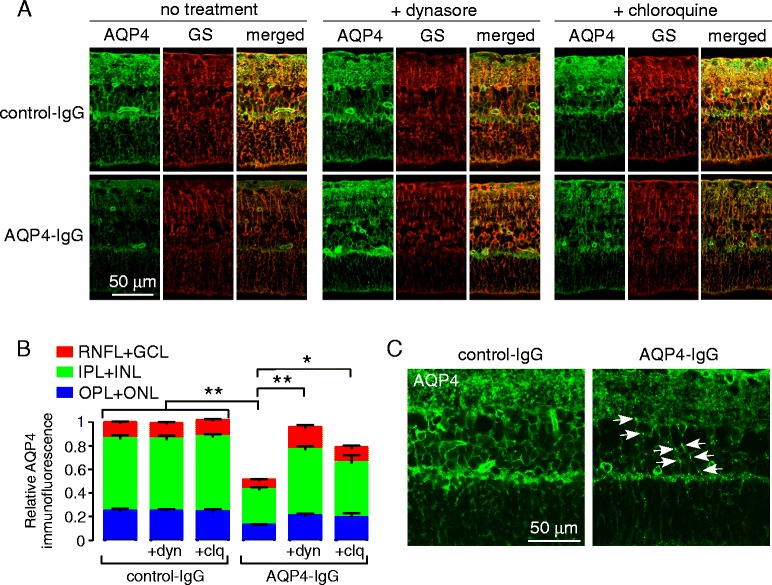
AQP4-IgG reduces Müller cell AQP4 expression in retinal explant cultures by an endocytosis mechanism. **a** AQP4 and GS immunofluorescence in retinal explant cultures treated with 20 μg/mL AQP4-IgG (or control-IgG) for 24 h, in the absence or presence of 50 μM dynasore hydrate (dyn) or 10 μM chloroquine (clq). **b** Relative AQP4 immunofluorescence for experiments as in (**a**) (mean ± S.E.M, *n* = 6, *p < 0.05, **p < 0.01). **c** AQP4 immunofluorescence at high magnification for explant cultures treated for 4 h with AQP4-IgG (or control-IgG). *White arrows* indicate endocytic vesicles

Retinal explant cultures were used to investigate the mechanism of AQP4-IgG-induced AQP4 loss in Müller cells. Culture of untreated retinas in serum-free media enabled preservation of retinal structures and Müller cell AQP4 expression after 24 h in culture. Subsequent incubation with AQP4-IgG, without added complement or leukocytes, produced marked, ~50 % reduction in Müller cell AQP4 expression 24 h later (Fig. 6a, b). To investigate whether an endocytosis mechanism might be responsible for the reduced AQP4 expression, the retinas were incubated during the 24-h exposure to AQP4-IgG with an inhibitor of clathrin-mediated endocytosis, dynasore, or an inhibitor of lysosomal acidification/degradation, chloroquine. AQP4-IgG-induced AQP4 loss was largely prevented by dynasore or chloroquine (Fig. 6a, b). High-magnification confocal microscopy of retinas at 4 h after addition of AQP4-IgG showed numerous AQP4^+^ puncta that were not seen in the control retinas, supporting an endocytic retrieval mechanism. Together, these data support an endocytosis and lysosomal degradation mechanism for AQP4 loss on AQP4-IgG-exposed Müller cells.

## Discussion

We found that exposure of retinal Müller cells to AQP4-IgG can produce primary retinal pathology in the absence of NMO optic neuritis. For these studies, we administered a purified, recombinant AQP4-IgG to rat eyes by an intravitreal route. AQP4-IgG diffused through the posterior segment where it accessed the ciliary epithelium and retinal Müller cells, without significant diffusion to AQP4 on retrobulbar optic nerve astrocytes. We found that intravitreal injection of AQP4-IgG produced pathology with loss of Müller cell AQP4, a gliotic response with increased GFAP, microglial activation with minimal leukocyte infiltration, and mild RGC loss and thinning of the GCC. An interesting and unexpected observation was that the changes were complement independent, which contrasts with the generally accepted NMO pathogenesis mechanism in the optic nerve, spinal cord, and brain. These findings have potential implications for pathogenesis mechanisms and therapy of retinal abnormalities in NMO.

The reduced Müller cell AQP4 expression following exposure to AQP4-IgG in ex vivo retinal cultures supports an endocytic retrieval mechanism with lysosomal targeting to account for the loss of AQP4. The comparable reduction in Müller cell AQP4 following intravitreal administration of an engineered AQP4-IgG lacking complement and cellular effector functions supports the conclusion that AQP4-IgG itself causes AQP4 internalization, as do the retinal culture studies in which neither complement nor effector cells were added. AQP4-IgG-induced internalization of AQP4 is likely cell-type-, polarization-, and, perhaps, even species-specific. While AQP4-IgG produces rapid AQP4 internalization in several AQP4-transfected cell lines, and moderately fast AQP4 internalization in nonpolarized astrocyte primary cultures, little AQP4-IgG-dependent AQP4 internalization was seen in astrocytes in the mouse brain in vivo [23, 24]. Though Müller cell AQP4 expression is polarized, the biology of Müller cells is different from that of astrocytes in the brain, spinal cord, and optic nerve. It is not clear from the studies here whether the loss of RGCs is a consequence of the loss of Müller cell AQP4 expression, the gliotic response, and/or intrinsic retinal inflammation.

The largely complement-independent retinal pathology following intravitreal AQP4-IgG administration contrasts with that seen in other CNS tissues. In humans, centrovascular deposition of activated complement is a characteristic pathological feature in NMO [25], and an initial open-label clinical study of a complement inhibitor showed efficacy in reducing attacks of NMO optic neuritis and transverse myelitis in seropositive NMO patients [26]. Pathology following passive transfer of AQP4-IgG to the mouse and rat brain, optic nerve, and spinal cord is predominantly complement dependent, as omission of added complement to mice [27–29] or inactivation of endogenous complement in rats [18, 19] prevents the development of NMO pathology. The complement-independent retinal injury found here suggests that complement-targeted therapeutics may have limited efficacy in some NMO patients with anterior visual pathway involvement.

Another interesting observation was increased GFAP expression in Müller cells following intravitreal AQP4-IgG administration, indicating a prominent gliotic response. Müller cell gliosis may be a consequence of intraretinal inflammation, Müller cell injury, and/or AQP4 loss. The retinal pathology seen here of AQP4 loss with increased GFAP and with minimal early complement deposition corresponds to the pathology seen in active human NMO lesions found in the brain and spinal cord classified as type 4 pathology [30]. Similarly, two separate models of chronic intrathecal AQP4-IgG infusion have shown loss of AQP4 expression in the spinal cord with reactive gliosis in the absence of complement activation [31, 32]. Thus, under conditions in which AQP4-IgG does not activate complement, the astrocytic response can be associated with GFAP upregulation rather than downregulation as seen when complement is activated. Release of soluble factors by activated microglia and Müller cells such as the proinflammatory cytokines TNF and monocyte chemoattractant protein MCP-1, which are thought to contribute to retinal degeneration in diabetic and other retinopathies [33], may contribute to the retinal pathology. Chronic functional loss of AQP4 water permeability in Müller cells was found in AQP4 knockout mice to produce abnormalities in retinal signal transduction as seen by electroretinography [34], which may reflect abnormal potassium and glutamate homeostasis. It is not clear whether the Müller cell gliosis and RGC loss produced by intravitreal AQP4-IgG is due to acute and partial loss of Müller cell water-transporting function.

Though the AQP4-expressing ciliary epithelium strongly bound AQP4-IgG following intravitreal AQP4-IgG administration, no pathology was seen at the light microscopic level. This is consistent with the near absence, albeit for a few case reports of NMO-related myositis [35, 36], of NMO pathology in peripheral organs in which AQP4 is expressed, including the skeletal muscle, stomach, kidney, airways, and exocrine glands. High-dose systemic administration of AQP4-IgG to mice or rats produces prompt and extensive AQP4-IgG deposition on peripheral AQP4-expressing organs, but with no pathology [18, 37]. Humans have been reported with circulating AQP4-IgG for at least a decade preceding clinical NMO disease [38]. Why peripheral, AQP4-expressing tissues are not damaged in seropositive NMO, despite their exposure to AQP4-IgG, remains unclear. It has been speculated that the unique cellular and physical environment in the CNS may be responsible for the development of NMO pathology in CNS but not peripheral tissues, as might the presence of complement inhibitor or other anti-inflammatory mechanisms in peripheral tissues.

During the completion of our manuscript, a paper by Zeka et al. [39] was published reporting that rats receiving AQP4-specific T cells developed retinitis with T cell infiltration and axonal pathology and that loss of AQP4 on Müller cells was seen when AQP4-IgG was delivered systemically along with the AQP4-specific T cells. Whether the retinal changes were primary or secondary to optic nerve pathology is unclear, as is its relevance to retinal abnormalities in human NMO.

Though our study demonstrates that exposure of the retina to AQP4-IgG can produce primary retinal injury with associated RGC loss, it is not known whether such a mechanism occurs in human NMO. Whether AQP4-IgG can access and bind to AQP4 on Müller cells beyond the blood-retinal barrier has not been established. There are no reports to our knowledge of retinal abnormalities in NMO without a history of optic neuritis, making it difficult to resolve primary vs. secondary AQP4-IgG-induced retinal injury in human NMO. Retinal pathology has been identified in postmortem tissue of patients with NMO, with mild loss of calbindin-positive horizontal cells and moderate loss of Müller cells and scattered loss of AQP4 immunoreactivity [40]. There were also Iba1^+^ microglia in the inner retina with few CD45^+^ cells and little complement deposition. Our animal model recapitulates many of these features of retinal pathology in human NMO.

## Conclusions

Passive transfer of NMO anti-AQP4 autoantibody by intravitreal injection in rat eyes produced marked loss of Müller cell AQP4 expression with a gliotic response, microglial activation, and mild RGC loss. The action of the autoantibody was largely complement independent, which contrasts with NMO pathogenesis mechanisms in the brain, spinal cord, and optic nerve. The results here provide a potential explanation and mechanism for the retinal pathology seen in seropositive NMO.

## Abbreviations

AQP4: Aquaporin-4
AQP4-IgG: anti-AQP4 autoantibody
AQP4-IgG^-CDC^: Aquaporumab, AQP4-IgG-lacking effector functions
clq: Chloroquine
CNS: Central nervous system
control-IgG: Control human IgG
CVF: Cobra venom factor
dyn: Dynasore hydrate
GCC: Ganglion cell complex
GCL: Ganglion cell layer
GFAP: Glial fibrillary acidic protein
GS: Glutamine synthetase
H&E: Hematoxylin and eosin
HBSS: Hank’s balanced salt solution
Iba1: Ionized calcium-binding adaptor molecule-1
INL: Inner nuclear layer
IPL: Inner plexiform layer
LPS: Lipopolysaccharide
Neurobasal A: Neuronal growth medium
NMO: Neuromyelitis optica
ONL: Outer nuclear layer
OPL: Outer plexiform layer
PBS: Phosphate-buffered saline
RGCs: Retinal ganglion cells
RNFL: Retinal nerve fiber layer
SD-OCT: Spectral-domain optical coherence tomography
